# The impact of moral injury on the wellbeing of UK military veterans

**DOI:** 10.1186/s40359-021-00578-7

**Published:** 2021-05-05

**Authors:** Victoria Williamson, Dominic Murphy, Sharon A. M. Stevelink, Shannon Allen, Edgar Jones, Neil Greenberg

**Affiliations:** 1 King’s Centre for Military Health Research, Institute of Psychology, Psychiatry and Neuroscience, King’s College London, 10 Cutcombe Road, London, SE5 9RJ UK; 2 Combat Stress, Research Department, Tyrwhitt House, Combat Stress, Leatherhead, Surrey UK; 3 Department of Psychological Medicine, Institute of Psychology, Psychiatry and Neuroscience, King’s College London, 16 De Crespigny Park, London, SE5 8AF UK; 4 Department of Experimental Psychology, Anna Watts Building, University of Oxford, Oxford, OX2 6 GG UK

**Keywords:** Moral injury, PTSD, Suicidal ideation, Military, Veteran

## Abstract

**Background:**

Experiences of potentially morally injurious events (PMIEs) have been found to negatively impact the mental health of US personnel/veterans, yet little is known about the effect of PMIEs on the mental health of the UK Armed Forces (AF). This cross-sectional study aimed to examine the association between PMIEs and the mental health outcomes of UK AF veterans.

**Method:**

Assessments of PMIE exposure and self-report measures of common mental disorders were administered using an online questionnaire to 204 UK veterans. Subjects were classified as having experienced a morally injurious event (n = 66), a non-morally injurious traumatic event (n = 57), a ‘mixed’ event (n = 31), or no event (n = 50).

**Results:**

Potentially morally injurious experiences were associated with adverse mental health outcomes, including likely anxiety disorders and suicidal ideation, compared to those who reported no event exposure. The likelihood of meeting criteria for probable PTSD was greatest in those who had experienced a non-morally injurious trauma. No statistically significant association between alcohol misuse and experiencing a PMIE or traumatic event was observed.

**Conclusions:**

The results provide preliminary evidence that potentially morally injurious experiences are associated with adverse mental health outcomes in UK AF veterans. Further work is needed to better understand the interplay between morally injurious events and threat-based trauma in order to design effective pathways for prevention and intervention for people exposed to highly challenging events.

## Background

Military service can involve exposure to potentially morally injurious events (PMIEs), which are experiences that violate one’s moral or ethical code [1, 2]. PMIEs are understood to generally include acts of commission, omission or betrayal by trusted others [3]. More recent evidence suggests moral injury can follow ‘*mixed’* events which combine ethically challenging and more routine traumatic events, involving threatened or actual death, serious injury or sexual assault, which are otherwise consistent with criterion A for DSM-5 posttraumatic stress disorder (PTSD) [2, 4–6]. PMIEs in a military context are not uncommon; for example, 24% of US Marines reported engaging in at least one morally transgressive act and 28% experienced a betrayal event during deployment [7].

While moral injury is not currently considered a mental disorder, experiences of PMIEs can lead to negative attributions about the self (e.g. ‘I am an awful person’) and others, as well as profound feelings of guilt, anger, worthlessness and shame [8]. This in turn can lead to the development of formal psychological difficulties. Moral injury has been found to be significantly associated with PTSD, depression and suicidal ideation [9, 10]. Furthermore, patients who seek treatment for PTSD often report PMIEs as the most distressing event for them [5].

The majority of evidence relating to the impact of moral injury on wellbeing stems from studies carried out in US military or veteran populations [11, 12]. However, US and UK militaries often differ in terms of demographics (e.g. US troops are frequently younger, larger proportion of National Guards/reserves [13]), have distinct deployment experiences (e.g. US troops often deploy for longer) and there are differences in the rules of engagement which may affect exposure to PMIEs. Similarly, once personnel have left service, veterans in the UK and US may face a number of distinct challenges, including different experiences in accessing healthcare (e.g. National Health Service (NHS) versus Department of Veterans Affairs). Qualitative studies of UK veterans have shown that they experience intense psychological distress following a PMIE, which can significantly disrupt their daily functioning [2, 14]. However, the association between PMIE exposure and mental health outcomes in a UK veteran population has not yet been explored. Previous US military and veteran studies have shown a significant association between experiences of moral injury and likely mental disorders, including PTSD, anxiety and depression [3, 15, 16]. Whether the same occurs in a UK context is unknown.

To address this gap, the primary aim of this study was to examine the association between potentially morally injurious experiences and the mental health outcomes of UK Armed Forces (AF) veterans. We hypothesised that those exposed to morally injurious events or traumatic events would be more likely to report poor mental health outcomes, including PTSD, alcohol misuse, suicidality, depression and anxiety, than those who did not report exposure to a challenging event during the course of their military service.

## Methods

This study received ethical approval from King’s College London Research Ethics Committee (RESCM-17/18-4002).

### Participants

Between November 2018 and April 2019, eligible veterans (defined as serving personnel who had left the UK AF) were recruited to the study. Eligibility required veterans to be aged 18 years and above, English speaking, and to be willing to self-report their experiences during military service. No limitations on eligibility according to demographic characteristics (e.g. gender, age, rank, years of service) were imposed. Further, we did not restrict participation by deployment location or AF service branch.

We used opportunity sampling and recruited participants by circulation of the study information posted on social media, online platforms, veteran affiliated charities, veteran specific newsletters, and military-affiliated magazines. The sampling method of snowballing was also utilised with participants asked to share study information with other potentially eligible individuals. Individuals were screened for eligibility in line with study inclusion/exclusion criteria using self-report questions, with written informed consent obtained.

### Assessments

The online questionnaire was distributed via the internet using a secure server. The anonymity of subjects was protected as they were not asked to provide any personally identifying information in order to participate (e.g. name, home address). Prior to distribution, the questionnaire was piloted with UK AF veterans (n = 4, not included in the final sample) with questions and formatting adjusted accordingly. Basic demographic information (e.g. age, service branch, sex, etc.) was collected from participants.

To determine whether a participant had experienced a moral injury, all participants were asked whether or not they had experienced a challenging event during military service (i.e. “during your military service, did you ever experience an event that was a serious challenge to your sense of who you are, your sense of the world, or your sense of right and wrong?”). Veterans were asked to write a brief description of the event which was later classified by two independent researchers as a moral injury, a non-morally injurious trauma, or a ‘mixed’ event. This classification process is described in-depth by Williamson et al., (2020). Any UK AF veterans who self-reported having not experienced a challenging military event were classified as ‘no event’. The inclusion of veterans who did and did not self-report having exposure to events which challenged their ethical or moral code allowed for comparisons between groups.

#### Psychological assessment measures

Probable PTSD was assessed via the PCL-5 (cut off score = 33) [17], probable depression via the PHQ-9 (cut off score = 15) [18], probable anxiety disorder via the GAD-7 (cut off score = 8) [19], probable alcohol misuse via the AUDIT (cut off score = 16) [20], and probable suicidal ideation via the SBQ-R (cut off score = 7) [21]. Combat exposure was assessed via the Combat Exposure Scale [22] with a score between 17 and 41 indicative of moderate-high combat exposure. All measures have been widely used in military samples and found to have good reliability and validity [3, 23–26].

### Data analysis

Descriptive analyses were conducted to provide an overview of the sample characteristics (Table 1). Prior to the main analysis, data were screened for inaccuracies in data entry, missing values and the presence of outliers. Where more than 50% of data was missing, the participant was excluded from the analysis. This resulted in three subjects being excluded from the analysis. Consistent with previous studies [23], for the remaining participants, we replaced missing values in the psychometric scales with the lowest possible value if 25% or fewer items were missing. If more than 25% of items were missing, the response was counted as missing. Common Mental Disorders (CMD) reflects participants who met case criteria on the GAD, PHQ-9 and/or PCL-5. Chi^2^ and Fishers’ Exact tests (where the sample size was limited) were used to determine whether statistically significant differences between groups existed, with p values < 0.05 used to indicate statistical significance. Where appropriate, odds ratios (OR) or adjusted odds ratios (AOR) and 95% confidence intervals (CI) were calculated using logistic regression. AOR were adjusted for sex and age. The reference category was reporting no exposure to a challenging event during military service. Effect sizes were considered statistically significant if the 95% CI did not include 1.

**Table 1:** Participant demographic characteristics

	Moral injury	Trauma	Mixed	No event	*P*
N	66	57	31	50	
Age, M (SD)	50.0 (10.3)	50.4 (11.4)	51.3 (10.4)	52.1 (12.9)	0.36
Male, n(%)	58 (87.8)	51 (92.7)	27 (90.0)	41 (82.0)	0.39
Marital status, n(%)					0.41
Single, never married	5 (7.6)	3 (5.3)	4 (12.9)	8 (16.0)	
In a relationship	50 (75.7)	42 (73.7)	21 (67.7)	37 (74.0)	
Divorced/separated/widowed	11 (16.7)	12 (21.1)	6 (19.4)	5 (10.0)	
Education attainment, n(%)					0.38
School until ≤ 18 years	15 (23.1)	16 (28.1)	8 (25.8)	15 (30.0)	
Further education	21 (32.3)	12 (21.1)	8 (25.8)	12 (24.0)	
Higher education BSc	14 (21.5)	23 (40.4)	11 (35.5)	13 (26.0)	
Masters/Doctoral degree	15 (23.1)	6 (10.5)	4 (12.9)	10 (20.0)	
Suicidal ideation caseness	37 (56.1)	36 (63.2)	20 (64.5)	16 (32.0)	0.004
Met case criteria, n(%)					
PTSD	36 (54.6)	38 (66.7)	19 (61.3)	12 (24.0)	< 0.001
Alcohol misuse	19 (28.8)	14 (24.6)	7 (22.6)	9 (18.0)	0.60
Depression	21 (31.8)	28 (49.1)	15 (48.4)	11 (22.0)	0.01
Anxiety	34 (51.5)	36 (63.2)	20 (64.5)	11 (22.0)	< 0.001
CMD	43 (65.2)	41 (71.9)	22 (71.0)	14 (28.0)	< 0.001

No event = veteran did not report experiencing a challenging event during military service. PTSD = meets diagnostic criteria for likely PTSD on the PTSD Checklist for DSM-5 (PCL-5). Depression = meets diagnostic criteria for likely severe depression on the Patient Health Questionnaire (PHQ-9). Anxiety = meets diagnostic criteria for likely moderate anxiety on the Generalised Anxiety Disorder Checklist (GAD-7). Suicidal ideation = meets criteria for suicidal ideation on the Suicide Behaviours Questionnaire Revised (SBQ-R). CMD = common mental disorders, includes participants meeting case criteria on the GAD, PHQ-9 and/or PCL-5. P = refers to whether differences between veterans exposed to an event type exist (*p* < 0.05), examined via chi^2^ or fishers’ exact tests

## Results

### Demographic information

Participant demographic information can be found in Tables 1 and 2. Overall, the 204 veterans had served between 18 months to 42 years in the AF (mean = 17.3 years, SD 9.61). The majority were White British (n = 200, 98.2%) and 88.6% were male (n = 177). In terms of event exposure, 66 (33.4%) veterans self-reported exposure to an event that was classified as morally injurious, 57 (27.9%;) veterans reported exposure to non-morally injurious trauma (e.g. being under enemy fire, exposure to an explosion), 31 (15.2%) veterans reported a ‘mixed’ event, and 50 (24.5%) veterans did not report exposure to a challenging event during military service. As seen in Table 1, a substantial proportion of veterans across the sample met case criteria for probable PTSD, alcohol misuse, depression and anxiety disorders. High rates of suicidal ideation were also found. It should be noted that these are not prevalence rates or representative of mental disorder prevalence in the AF and may reflect the purposive sampling strategy used.

**Table 2:** Participant military-related demographic characteristics

	Moral injury	Trauma	Mixed	No event	*P*
N	66	57	31	50	
Branch, n(%)					0.98
Royal Navy/ Royal Marines	10 (15.15)	7 (12.5)	6 (19.4)	7 (14.0)	
Army	46 (69.7)	42 (75.0)	21 (67.7)	35 (70.0)	
Royal Air Force	10 (15.2)	7 (12.5)	4 (12.9)	8 (16.0)	
Rank, n(%)^a^					0.62
Officer	18 (29.0)	8 (14.6)	8 (27.6)	14 (28.6)	
Non-commissioned officer	20 (32.3)	21 (38.2)	9 (31.0)	17 (34.7)	
Junior rank	24 (38.7)	26 (47.3)	12 (41.4)	18 (36.7)	
Length of service (years), M (SD)	17.6 (9.6)	17.2 (9.1)	16.0 (8.5)	18.5 (10.5)	0.50
Deployed, n(%)	57 (86.4)	51 (89.5)	29 (96.7)	41 (83.7)	0.35
Number of deployments, M (SD)	5.0 (3.8)	7.9 (14.0)	5.1 (4.5)	4.5 (3.6)	0.29
Moderate-heavy combat exposure	32 (48.5)	31 (54.4)	16 (51.6)	18 (36.0)	0.27
Deployment theatre, n(%)^b^					
Iraq	29 (43.9)	28 (49.1)	18 (58.1)	21 (42.9)	0.53
Afghanistan	31 (47.0)	20 (35.1)	13 (41.9)	19 (38.0)	0.58
Bosnia/Kosovo	27 (40.9)	20 (35.1)	12 (38.7)	10 (20.0)	0.10
Other location	51 (79.7)	44 (78.6)	20 (64.5)	30 (62.5)	0.11
Deployment type, n(%)^b^					
Disaster assistance	13 (20.3)	7 (12.5)	4 (12.9)	4 (9.1)	0.42
Peacekeeping	29 (45.3)	21 (37.5)	13 (41.9)	19 (43.2)	0.86
Peace enforcement	26 (40.6)	24 (42.9)	12 (38.7)	16 (36.4)	0.93
Warfighting	41 (64.1)	38 (67.9)	19 (61.3)	24 (54.6)	0.58
Other duty	9 (14.1)	8 (14.3)	7 (22.6)	8 (18.2)	0.69

No event = veteran did not report experiencing a challenging event during military service. Deployed = reported having been on a deployment. a = data missing for 10 veterans. b = individuals could report deployment to multiple deployment types and theatres. Moderate – heavy combat exposure = Combat Exposure Scale (CES) score between 17 and 41. P = refers to whether differences between veterans exposed to an event type exist (p < 0.05), examined via chi^2^ or fisher’s exact tests

No statistically significant differences were found between exposure groups in terms of socio-demographic or military-related characteristics (see Tables 1 and 2). Many veterans across the sample reported a high level of education attainment and a considerable proportion were officers or non-commissioned officers (n = 123, 57.7%). No significant differences were found between deployment location, extent of combat exposure or number of deployments and exposure to morally injurious, traumatic or ‘mixed’ events (Table 2).

### Relationship between type of event exposure and mental health

Only adjusted analysis will be discussed. Exposure to specific types of challenging events in military service was found to be associated with a range of adverse mental health outcomes. Compared to veterans who did not report exposure to a challenging event during military service, veterans who experienced a morally injurious event (AOR 3.98; 95% CI 1.75–9.05) were significantly more likely to meet case criteria for probable PTSD (see Table 3), anxiety (AOR 3.91; 95% CI 1.69–9.04) and suicidal ideation (AOR 2.60; 95% CI 1.20–5.66) compared to those who did not report a challenging event during military service. Compared to those who reported no challenging event during military service, veterans who were classified as having experienced a ‘mixed’ event were also more likely to meet criteria for likely PTSD (AOR 5.21; 95% CI 1.93–14.06; Table 3), depression (AOR 3.36; 95% CI 1.25–9.01), anxiety (AOR 6.75; 95% CI 2.44–18.72), and suicidal ideation (AOR 3.89; 95% CI 1.48–10.21). Veterans who reported exposure to a traumatic, non-morally injurious event were more likely to meet case criteria for probable PTSD (AOR 6.67; 95% CI 2.77–16.07), depression (AOR 3.38; 95% CI 1.41–8.05), anxiety (AOR 6.29; 95% CI 2.59–15.25) and suicidal ideation (AOR 3.33; 95% CI 1.47–7.58) compared to those who did not report a challenging event during military service.

**Table 3:** Likely mental disorders in veterans exposed to morally injurious, traumatic and mixed events (reference category = no event)

	Moral injury	Trauma	Mixed
PTSD			
OR (95%CI)	**3.61 (1.63–7.97)**	**5.85 (2.54–13.50)**	**4.87 (1.88–12.64)**
AOR (95%CI)	**3.98 (1.75–9.05)**	**6.67 (2.77–16.07)**	**5.21 (1.93–14.06)**
Alcohol misuse			
OR (95%CI)	1.74 (0.73**–**4.15)	1.33 (0.53**–**3.34)	1.37 (0.47**–**3.93)
AOR (95%CI)	1.89 (0.77**–**4.66)	1.35 (0.52**–**3.54)	1.55 (0.52**–**4.60)
Depression			
OR (95%CI)	1.59 (0.70**–**3.62)	**3.14 (1.37–7.19)**	**3.24 (1.26–8.39)**
AOR (95%CI)	1.71 (0.72**–**3.99)	**3.38 (1.41–8.05)**	**3.36 (1.25–9.01)**
Anxiety			
OR (95%CI)	**3.55 (1.58–7.95)**	**5.57 (2.40–12.92)**	**6.20 (2.34–16.45)**
AOR (95%CI)	**3.91 (1.69–9.04)**	**6.29 (2.59–15.25)**	**6.75 (2.44–18.72)**
Suicidal ideation			
OR (95%CI)	**2.47 (1.16–5.25)**	**3.43 (1.55–7.58)**	**3.82 (1.50–9.71)**
AOR (95%CI)	**2.60 (1.20–5.66)**	**3.33 (1.47–7.58)**	**3.89 (1.48–10.21)**

*OR* odds ratios. *AOR*  adjusted odds ratio for sex and age. For OR and AOR the reference category was ‘no event’. Suicidal ideation = scored above SBQR-R cut off score of 7 used to identify individuals at risk of suicide. Bold values denote statistical significance

Notably, veterans who reported a moral injurious, a ‘mixed’ or a traumatic, non-morally injurious event were not more likely to report alcohol misuse compared to those who reported no event exposure (see Table 3). Moreover, the likelihood of meeting case criteria for a probable anxiety disorder and suicidal ideation was greatest in the ‘mixed’ group, compared to those who reported morally injurious or non-morally injurious traumatic events (Table 3). The likelihood of meeting case criteria for probable PTSD and depression was greatest in those who had experienced a non-morally injurious trauma. Nonetheless, overlapping confidence intervals indicate these differences between exposure groups are not statistically significant but may indicate an upward trend.

## Discussion

This study aimed to investigate the impact of exposure to morally injurious events on the mental health of UK AF veterans. Three key findings were observed. First, experiences of moral injury were associated with adverse mental health outcomes, with veterans exposed to ‘mixed’ events having a greater likelihood of meeting criteria for probable anxiety and suicidal ideation. Secondly, the likelihood of meeting criteria for probable PTSD was greatest in those who had experienced a non-morally injurious trauma, although overlapping confidence intervals suggest these differences are not statistically significant. Thirdly, there was no statistically significant association between alcohol misuse and experiencing a PMIE or traumatic event.

The results of this study indicate that, as with incidents meeting the DSM-5 definition of a traumatic event [4], experiences of moral injury, trauma and ‘mixed’ events are significantly associated with poor mental health outcomes in the UK AF. The results are consistent with previous studies in the US militaries where experiences of morally injurious events are significantly associated with adverse mental health outcomes, including PTSD, anxiety and suicidal ideation [11, 12]. A meta-analysis highlighted the lack of non-US research into the impact of moral injury on mental health [9]. The present study addresses this gap by providing preliminary evidence of the detrimental effect of traumatic events, including PMIEs, on the mental health of UK veterans.

It is notable that the likelihood of meeting case criteria for probable anxiety and suicidal ideation was greatest in the ‘mixed’ group compared to those who reported moral injurious or non-morally injurious traumatic events. While this difference was not statistically significant (potentially due to the small sample size), this finding could indicate that those who experience events which are both traumatic and morally injurious may be at particular risk of suicidality which is consistent with previous qualitative studies [2, 5]. It is possible that the combined impact of both a traumatic and PMIE may act as a ‘double stressor’. These events could potentially complicate treatment as clinicians may be more likely to focus on the traumatic aspects of the event—rather than the morally injurious features—which are well addressed by conventional models of PTSD care. Therefore, these results tentatively contribute towards the conceptual clarification of moral injury in a UK context as well as having a practical application in that it is clear that clinicians taking a trauma history from veterans should specifically ask about potentially morally injurious aspects related to traumatic incidents. Where ‘mixed’ events are identified, our results suggest that particular attention should be paid to risk assessment for suicidality. As suggested by previous studies [27], standard exposure-based treatments for PTSD may not adequately address the needs of those who are suffering after potentially morally injurious or ‘mixed’ events. Other approaches, such as Adaptive Disclosure or Acceptance and Commitment Therapy [28, 29], could potentially be more suitable in such cases to address patient’s maladaptive psychological responses of guilt and shame.

This study also found that the likelihood of meeting criteria for probable PTSD was somewhat greater in those who had experienced a non-morally injurious traumatic event. However, this finding was not significantly different to the lower rates found after ‘mixed’ and morally injurious incidents. Although, this differential profile of distress is consistent with previous research that participants who experience traumatic events which elicit fear (rather than moral crisis) report higher levels of hyperarousal/hypervigilance symptoms relative to those who report non-life threat traumas [30]. It is possible that the lack of statistically significant differences between PMIE and trauma exposed groups could reflect the opportunity sampling approach used, in that individuals who took part in the study may have been especially motivated to share their experience, causing a ceiling effect.

Interestingly, this study did not find an association between experiences of moral injury and/or trauma and alcohol misuse, which is inconsistent with some previous studies of war-related PMIEs [31]. This is a surprising finding as alcohol misuse has been found to be an avoidant coping strategy often used post-trauma [32]. However, it may be that heavier drinking is an endemic problem for the AF and such behaviours may mask drinking as an avoidance coping strategy. Future large-scale studies are needed to further examine the prevalence of mental health difficulties following PMIEs in UK veterans to better understand their occurrence, with direct comparisons made between the UK and other AF to advance our understanding of the psychological impact of moral injury.

This study has several strengths and weaknesses. Among the strengths is that participation was anonymous and confidential, which may have facilitated disclosure of veteran experiences and associated distress. That no veterans were excluded on the basis of demographic or military characteristics (e.g. AF branch, length of service or deployment location, etc.) is also a strength. Nonetheless, the cross-sectional design of this study prevents causal inferences and the convenience sampling strategy used may reduce the generalisability of the present findings. Another limitation of the study is that the details of military service and trauma exposure provided by subjects were not verified from official records, so we cannot be sure of their accuracy. The sample also was not representative of UK AF given the high percentage of officers and non-commissioned officers (NCOs) who took part in the study. There may be biases associated with a population that responds to advertisements and participant invitations in social media and military charities rather than by random selection. Additionally, the assignment of participants to moral injury, ‘mixed’ or trauma groups was determined by independent researcher ratings and future studies should use a screening measure once a validated tool for detecting moral injury is developed for use in the UK AF. Efforts are currently underway, both in the UK and internationally [33, 34], to develop a validated measure of moral injury exposure that may improve not only improve the identification of those suffering following moral injury but reduce bias in future studies. Finally, the presence of probable mental health problems was assessed via self-report questionnaire rather than clinical interview which is considered the gold-standard for mental disorder assessment.

## Conclusions

The current study provides the first preliminary evidence that experiences of moral injury are associated with adverse mental health outcomes in UK AF veterans. The findings also expand on the limited available research into the specific psychological impact of experiencing ‘mixed’ events that are simultaneously morally injurious and traumatic/threatening – a distinction that has not been clearly made before in the existing literature. That ‘mixed’ events may be associated with particular mental health difficulties, such as anxiety and suicidal ideation, stresses the importance of comprehensive trauma history taking in clinical settings. Finally, this research underscores the pressing need for a validated measure for moral injury appropriate for the UK AF to not only better understand the impact of moral injury on veteran wellbeing, but also to ensure that appropriate treatment can be provided to those identified as suffering post-trauma.

## Data Availability

No additional data are available.

## Abbreviations

PTSD: Posttraumatic stress disorder
PMIE: Potentially morally injurious events
AF: Armed Forces
NHS: National Health Service
